# Detection and quantification of SARS-CoV-2 by droplet digital PCR in real-time PCR negative nasopharyngeal swabs from suspected COVID-19 patients

**DOI:** 10.1371/journal.pone.0236311

**Published:** 2020-09-08

**Authors:** Claudia Alteri, Valeria Cento, Maria Antonello, Luna Colagrossi, Marco Merli, Nicola Ughi, Silvia Renica, Elisa Matarazzo, Federica Di Ruscio, Livia Tartaglione, Jacopo Colombo, Chiara Grimaldi, Stefania Carta, Alice Nava, Valentino Costabile, Chiara Baiguera, Daniela Campisi, Diana Fanti, Chiara Vismara, Roberto Fumagalli, Francesco Scaglione, Oscar Massimiliano Epis, Massimo Puoti, Carlo Federico Perno

**Affiliations:** 1 Department of Oncology and Hemato-oncology, University of Milan, Milan, Italy; 2 Residency in Microbiology and Virology, Università degli Studi di Milano, Milan, Italy; 3 Department of Laboratories, Bambino Gesù Children's Hospital, Rome, Italy; 4 Infectious Diseases, ASST Grande Ospedale Metropolitano Niguarda, Milan, Italy; 5 Rheumatology Unit, ASST Grande Ospedale Metropolitano Niguarda, Milan, Italy; 6 Department of Cardiotoracovascular Anesthesia and Intensive Care, ASST Grande Ospedale Metropolitano Niguarda, Milan, Italy; 7 Department of Laboratory Medicine, ASST Grande Ospedale Metropolitano Niguarda, Milan, Italy; 8 Department of Pathophysiology and Transplantation, University of Milan, Milan, Italy; 9 Department of Anesthesiology, Critical Care and Pain Medicine, ASST Grande Ospedale Metropolitano Niguarda, Milan, Italy; US Army Medical Research Institute of Infectious Diseases, UNITED STATES

## Abstract

Since SARS-CoV-2-based disease (COVID-19) spreads as a pandemic, the necessity of a highly sensitive molecular diagnosis that can drastically reduce false negatives reverse transcription PCR (rtPCR) results, raises as a major clinical need. Here we evaluated the performance of a ddPCR-based assay to quantify SARS-CoV-2 titer in 55 suspected COVID-19 cases with negative rtPCR results thanks to in-house ddPCR assay (targeting RdRp and host RNaseP). Samples were collected at ASST-GOM Niguarda between February and May 2020 at hospital admission. Clinical and imaging data were obtained for clinical staging and definition of disease severity. Patients were mainly female (45.5%) with a median age of 73 (57–84) years. ddPCR-based assay detected SARS-CoV-2 genome in nasopharyngeal samples of 19 (34.5%) patients (median viral-load: 128 copies/mL, IQR: 72–345). In 15 of them (78.9%), chest CT showed a classical COVID-19 bilateral interstitial pneumonia; 14 patients (73.7%) showed severe COVID-19 manifestations. ddPCR did not identify any trace of SARS-CoV-2 genome in the respiratory samples of the remaining 36 patients. The serological assay performed in a subgroup of 34 patients at the later stage of illness (from 3 days to 90 days after) confirmed the presence of SARS-CoV-2 antibodies in all patients tested positive for SARS-CoV-2 in ddPCR (100%). Contrariwise, negative tests were observed in 95.0% ddPCR negative patients (P<0.001). Thanks to a ddPCR-based assay, we achieved a rapid and accurate SARS-CoV-2 diagnosis in rtPCR-negative respiratory samples of individuals with COVID-19 suspect, allowing the rapid taking care and correct management of these patients.

## Introduction

The pandemic of COVID-19, caused by severe acute respiratory syndrome coronavirus 2 (SARS-CoV-2), has posed a serious threat to global public health, calling for the development of reliable diagnostic tests, able to identify (and possibly quantify) SARS-CoV-2. Currently recommended diagnostic tests for SARS-CoV-2 detection are real-time PCR (rtPCR) assays [1, 2], which are able to detect viral RNA through the amplification of 2 or 3 distinct genomic regions.

Despite the availability of these methods, the diagnostic optimum is far to be reached. The dynamic range of these tests is limited, and their diagnostic sensitivity on nasopharyngeal and oropharyngeal swabs has been shown to be insufficient in a number of SARS-CoV-2 related pneumonia cases [3–5]. First evidences suggest false negative results in 20%-30% of cases [3,6]. The persistently negative rtPCR results in patients with clinical suspects of COVID-19, and no alternative diagnosis, could be related to the lower viral titers that characterize the most widely used nasopharyngeal samples [7] in comparison to other respiratory specimens, such as sputum [8], as well as to the replication kinetics of the virus. Initial data are indeed suggesting that viral loads in throat swab and sputum samples peak at around 5–6 days after symptoms onset [9], with consequent risk of uncontrolled SARS-CoV-2 transmission in the time period preceding the viral load peak.

The prompt diagnosis of COVID-19 patients is therefore a clinical need that is only partially met, which advocates for more sensitive and accurate diagnostic technologies.

Droplet Digital PCR (ddPCR) is a highly sensitive assay for the direct detection and quantification of DNA and RNA targets. It has been increasingly used in infectious disease settings, especially thanks to its ability to consistently and reliably detect down to few copies of viral genomes [10–14]. Whether the detection of low-level and/or residual viral presence is required, quantitative data obtained by ddPCR are far more informative than those provided by standard rtPCR assays [13]. Standing the necessity of a limitation (as much as possible) of false negative results in COVID-19 diagnosis, the use of ddPCR could provide a critical support [8]. Its use for SARS-CoV-2 detection, however, is still very poorly investigated in clinical settings, and no data are currently available for European patients.

In this study, the presence of SARS-CoV-2 genome was evaluated in 55 SARS-CoV-2 rtPCR negative nasopharyngeal swabs from COVID-19 suspected patients thanks to a quantitative *ad hoc* designed assay based on ddPCR.

## Material and methods

### Clinical sample collection

Negative nasopharyngeal swabs tested for SARS-CoV-2 by rtPCR (GeneFinder^TM^ COVID-19 Plus RealAmp Kit, ELITech; Allplex^TM^ 2019-nCoV Assay, Seegene) were collected during February-April 2020 from 55 COVID-19 suspected patients at ASST GOM Niguarda admission. For each patient, demographic and clinical information such as age, gender, clinical manifestations and symptoms were retrieved and stored in an anonymous database ad hoc built for the study. The severity of the COVID-19 was classified into mild, moderate, or severe, if showing i) mild clinical symptoms without sign of pneumonia on imaging, ii) fever and respiratory symptoms with radiological findings of pneumonia, iii) respiratory distress, with oxygen saturation ≤93% at rest, mechanical ventilation, or presence of multiorgan failure (septic shock) and/or admission to intensive care unit (ICU) hospitalization.

### Ethical committee

The authors confirm that the ethical policies of the journal, as noted on the journal’s author guidelines page, have been adhered to. Medical records were registered and processed by using pseudonymization measures and assuring that it was not (and it is no longer) permitted the identification of data subjects. The study protocol was approved by the ASST Grande Ospedale Metropolitano Niguarda Research Ethics Committee (prot. 92–15032020). Signed informed content was obtained for each participant. This study was conducted in accordance with the principles of the 1964 Declaration of Helsinki.

### SARS-CoV-2 quantification by ddPCR

Total RNA was extracted from 280 ul of nasopharyngeal swabs using QIAamp viral RNA mini kit (Qiagen) following manufacturer’s instruction. SARS-CoV-2 genomic RNA was quantified by means of the QX200™ Droplet Digital™ PCR System (ddPCR, Biorad) using an home-made protocol targeting the RNA dependent RNA polymerase (RdRp) of SARS-CoV-2 (Forward: 5’- GACTTTGTGAATGAGTTTTACGC-3’, Reverse: 5’- AGCCACTAGACCTTGAGATGC-3’ and FAM Probe: 5’- CACACAACAGCATCGTCAGA-3’) and the housekeeping gene RNAse P [15] HEX (Forward: 5’- AGATTTGGACCTGCGAGCG-3’, Reverse: 5’- GAGCGGCTGTCTCCACAAGT -3’ and HEX Probe: 5’- TTCTGACCTGAAGGCTCTGCGCG-3’). The cycling conditions were: 45°C (60 min), 95°C (10 min), 40 cycles of 95°C (30 sec) and 58°C (1 min), 98°C (10 min), 4°C (∞). SARS-CoV-2 quantification was finally expressed in number copies/mL of swab.

### Limit of detection of ddPCR assay

To verify the correct performance of the SARS-CoV-2 RNA quantification and housekeeping gene quantification, two independent experiments using different dilutions of the SARS-CoV-2 RNA from a reference patient (rtPCR cycle n. 20 corresponding to 10^7^ copies/mL in ddPCR) were performed. In particular, six serial dilutions were performed in order to deposit 10^5^, 10^4^, 10^3^, 10^2^, 10, 2 copies per reaction. All samples were repeated in duplicate, with exception of the last point repeated in quadruplicate. Coefficient of determination (R^2^) of SARS-CoV-2 quantification was assessed by linear regression analysis by plotting the standards’ measured copies and comparing them with expected values of serial dilutions.

Negative and positive controls, consisting of 40 RNA samples obtained from human nasopharyngeal swabs collected before September 2019, and 60 RNA samples obtained from human SARS-CoV-2 positive nasopharyngeal swabs collected during the pandemics were included in each experiment. Negative samples for SARS-CoV-2 were retrospectively selected on the basis of their availability, the storage at -80°C in order to preserve eventual presence of RNA, and the collection date (September 2019-January 2019). An alternative confirmed diagnosis was available for most of these samples (Influenza A, n = 15; Bacterial infections, n = 9; Influenza B, n = 3, RSV, n = 2; HCov-OC43, n = 1).

### Serological assay

In order to further confirm the performance of the ddPCR-based assay, IgG against SARS-CoV-2 were tested by using a commercial Chemiluminescent microparticle immunoassay IgG against SARS-CoV-2 (https://www.corelaboratory.abbott/us/en/offerings/segments/infectious-disease/sars-cov-2, sensitivity:99.9% specificity:100%) [16] in a subgroup of 34 patients for those serum samples at the later stage of illness (from 3 days to 100 days after) were available (SARS-CoV-2 ddPCR positive: 14; SARS-CoV-2 ddPCR negative: 22).

### Statistical analyses

For ddPCR methods characterization, coefficient of determination (R2) was assessed by linear regression analysis, whereas the limit of detection (LoD), defined as the lowest concentration at which 95% of positive samples were detected, was determined by probit regression analysis. Reproducibility of SARS-CoV-2 quantification methods was assessed by intra- and inter-run tests using serial standard dilutions. The coefficient of variation (CV) was calculated as the standard deviation (SD) of SARS-CoV-2 copies/reaction divided by replicates mean. Descriptive statistics are expressed as median values and interquartile range (IQR) for continuous data and number (percentage) for categorical data. To assess significant differences, fisher exact test and Wilcoxon test were used for categorical and continuous variables, respectively. A p-value <0.05 was considered statistically significant. Statistical analyses were performed with SPSS software package for Windows (version 23.0, SPSS Inc., Chicago, IL).

## Results

### Study population

Baseline demographic and clinical characteristics of the 55 patients included in the study are reported in Table 1. Patients were mainly female (45.5%) with a median age of 73 (IQR: 57–84) years.

**Table 1:** Baseline demographic and clinical characteristics of the study population.

	Overall	Patients		p-value^a^
		SARS-CoV-2 positive	SARS-CoV-2 negative	
**Patients, N**	55	19	36	
**Males**	25 (45.5)	10 (52.6)	20 (55.6)	0.530
**Age (years)**	73 (57–84)	73 (60–84)	71 (57–84)	0.956
**Date of symptoms onset**	March 20 (March 5—April 26)	March 14 (March 1-March 25)	April 15 (March 9-April 28)	0.083
**Date of Hospital admission**	April 20 (March 11-April 29)	March 18 (March 8-April 4)	April 28 (March 9-April 28)	**0.010**
**Date of first nasopharingeal swab**	April 28 (March 13-May 01)	March 17 (March 8-April 7)	April 30 (April 9-April 28)	**0.003**
**Time from symptoms-onset to hospital admission, days**	4 (0–9)	4 (0–6)	4 (0–10)	0.425
**Time from symptoms-onset to nasopharingeal swab, days**	5 (2–10)	5 (1–6)	5 (2–14)	0.210
**Positivity to the serological assay**^**b**^	15 (44.1)	14 (100.0)	1^c^ (5.0)	**<0.001**
**Time from symptoms-onset to serological assay, days**^**b**^	17 (9–33)	22 (13–33)	16 (9–33)	0.495
**Symptoms at Hospital admission**				
*Fever*, *°C*	35 (63.6)	15 (78.9)	20 (55.6)	0.076
*Cough*	22 (40.0)	11 (57.9)	11 (30.6)	**0.047**
*Dyspnea*	30 (54.5)	17 (89.5)	13 (36.1)	**<0.001**
**Pulmonary involvement**	36 (65.5)	18 (94.7)	18 (50.0)	**0.001**
*Bilateral interstitial pneumonia*	16 (29.1)	15 (78.9)	1^d^ (2.8)	**<0.001**
*Pneumonia with pleuritis*	6 (10.9)	1 (5.3)	5 (13.9)	0.635
*Lung cancer*	2 (3.6)	1 (5.3)	1 (2.8)	0.986
*Lobar pneumonia*	6 (10.9)	1 (5.3)	5 (13.9)	0.635
*Pneumonia “ab ingestis”*	1 (1.8)	0 (0.0)	1 (2.8)	1.000
*Bacterial pneumonia*	3 (5.5)	0 (0.0)	3 (8.3)	0.544
*Fungal pneumonia*	1 (1.8)	0 (0.0)	1 (2.8)	1.000

COVID-19, Coronavirus Disease 2019. Data are expressed as median (interquartile range, IQR), or N (%).

^a^Fisher exact test and Wilcoxon test were used for categorical and continuous variables, respectively. Statistically significant p-values are in bold.

^b^ Available for 34 patients and tested by Chemiluminescent microparticle immunoassay IgG against SARS-CoV-2 (https://www.corelaboratory.abbott/us/en/offerings/segments/infectious-disease/sars-cov-2).

^c^Patient with a pneumonia with pleuritis, characterized by a time from symptoms-onset to nasopharingeal swab of 28 days, and a time from symptoms-onset to serological assay of 48 days.

^d^ Patient with a bilateral interstitial pneumonia, characterized by a time from symptoms-onset to nasopharingeal swab of 11 days, and repeatedly tested negative for SARS-CoV-2. Serological data are not available.

All patients were symptomatic with exception of one (COVID-19 contact) and thus immediately hospitalized and tested for SARS-CoV-2 rtPCR, whose result was negative. Thirty-five patients reported fever (63.6%) and 36 had pulmonary involvement (65.5%). Further chest CT showed a bilateral interstitial pneumonia in 16 patients (29.1%). For the remaining 20, other lung diseases were diagnosed (Table 1).

### Performance of the assays

**Fig 1:**
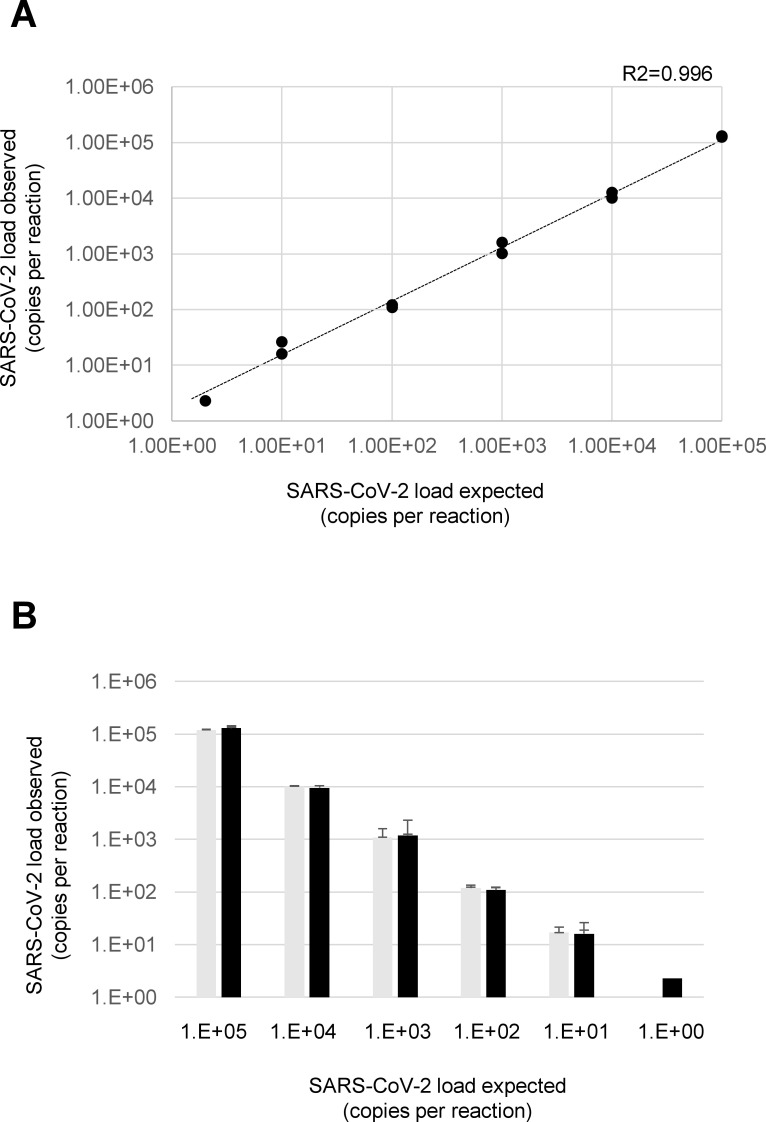
Quantification of SARS-CoV-2 by ddPCR. **A)** The graph shows the linear relationship between the expected and the observed concentrations using serial dilutions of the SARS-CoV-2 reference in the two experiments. **B)** The histogram reports the concentrations of the SARS-CoV-2 reference obtained in the first (light grey) and second (dark grey) experiment. In particular, each expected concentration was tested in two independent experiments each led in duplicate (the lowest concentration, 2 copies per reaction, led in quadruplicate).

The method showed a good linear correlation between expected and observed SARS-CoV-2 quantification (R2 = 0.996) (Fig 1A and S1A Fig).

By intra-run tests, the differences between the expected and observed SARS-CoV-2 copy number per reaction in the two experiments were 121,200±566 and 124,400±1,697 for 10^5^ copies, 10,080±113 and 12,560±2,715 for 10^4^ copies, 1,035±26 and 1,580±254 for 10^3^ copies, 108±11 and 120±3 for 10^2^ copies, 16±3 and 26±3 for 10 copies, 2.2±3.3 for 2 copies.

For inter-run tests, the mean (+SD) SARS-CoV-2 copy number per reaction was 122,800±2,262 for 10^5^ copies, 11,320±1,753 for 10^4^ copies, 1,307±385 for 10^3^ copies, 114±8 for 10^2^ copies, 21±7 for 10 copies, 1.1±1.5 for 2 copies (Fig 1B). Mean CV was 0.09 and 0.15 for intra-run and inter-run tests, respectively.

Probit analysis predicted a limit of detection (LoD) of 2.9 (95% IC 2.0–11.5) copies per reaction.

No signal was detected in any of the 40 SARS-CoV-2 negative samples tested, all collected between June and September 2019, before SARS-CoV-2 pandemic (S1B Fig). SARS-CoV-2 genome was quantified in all the 60 RNA samples obtained from human SARS-CoV-2 positive swabs included as positive controls (copies per reaction, median [IQR]: 665 [102–5,790], corresponding to a viral load of 39,900 [5,340–347,400] copies/mL).

### Detection of SARS-CoV-2 from pharyngeal swab samples

The rtPCR-negative nasopharyngeal swab samples obtained at first visit from the 55 patients included in the analysis were tested with ddPCR in blind. The quality of all swab samples was confirmed by RNAseP quantification (median, IQR, copies/mL: 136,286 [84,548–256,714]).

Our home-made ddPCR assay detected SARS-CoV-2 in nasopharyngeal swab samples belonged to 19 patients (Table 1 and S1 Table). Median viral load was 128 copies/mL (IQR: 72–345); the highest value detected was 1800 copies/mL (S1C and S1D Fig). Of note, for 12 patients the subsequent nasopharyngeal swabs gave multiple negative rtPCR results (median number of negative swabs: 3 [3–4]). For the remaining 7 patients SARS-CoV-2 was later on detected also by rtPCR, during the follow-up investigation performed within 3 days since the initial negative test. ddPCR provided negative results in the remaining 36 nasopharyngeal swabs.

Table 1 reported demographic and clinical characteristics respect to ddPCR results. Patients with a ddPCR confirmed SARS-CoV-2 infection were more recently admitted to the hospital (March 18 vs. April 28, P = 0.008), were more frequently affected by cough and dyspnea (P = 0.047 and <0.001, respectively), and showed more frequently a classical bilateral interstitial pneumonia (P<0.001) respect to patients with a negative ddPCR result. Of note, a severe COVID-19 manifestation characterized 14 of the 19 patients with a ddPCR confirmed SARS-CoV-2 infection (73.7%, S1 Table). Median time from symptoms-onset to hospital admission was not significantly different between the two groups (P = 0.425).

### Serological assays results

In order to further confirm the performance of the ddPCR-based assay in SARS-CoV-2 detection, IgG against SARS-CoV-2 were tested by using a commercial chemiluminescent immunoassay [16] in a subgroup of 34 patients for those serum samples at the later stage of illness (from 3 days to 90 days after) were available (SARS-CoV-2 positive: 14; SARS-CoV-2 negative: 20). In this subgroup of patients, antibodies were detected in all patients tested positive for SARS-CoV-2 in ddPCR (14/14). The SARS-CoV-2 RNA positivity in the 5 samples with a serological assay not available (ID 1, 4, 8, 12, 13) was further confirmed by using a second assay adapted for ddPCR and targeting two different portions of RdRp [17]. SARS-CoV-2 was detected at later time points also by standard qualitative rtPCR for three of these five patients (ID 1, 4, 8, S1 Table). Contrariwise, negative antibody findings were observed in 19/20 (95.0%) patients tested negative for SARS-CoV-2 in ddPCR (Table 1). The single patient tested negative for SARS-CoV-2 by ddPCR, but positive at the serological assay was characterized by a time from symptoms-onset to nasopharingeal swab of 28 days, and a time from symptoms-onset to serological assay of 48 days, thus suggesting a late-stage disease at hospital admission, when probably the viral clearance already occurred.

## Discussion

This proof-of-concept study shows that an in-house ddPCR-based assay can allow an efficient detection of SARS-CoV-2 at low copy number in symptomatic cases resulted negative by standard rtPCR. The effective prevention, treatment and control of COVID-19 cannot be achieved without an early, sensitive, and reliable diagnosis. The laboratories play a critical role in confirming the initial clinical suspicion of this disease, as confirmation of SARS-CoV-2 presence is essential to ensure the prompt initiation of containment and treatment protocols. This is of utmost importance to avoid further spread of the pandemic, and to assure the best clinical and therapeutic management of the infected patients in the hospital setting.

Unfortunately, currently used rtPCR assays lack of the necessary sensitivity to identify all cases of SARS-CoV-2 infection (20% of false negative results [5,6]). Complementary laboratory assays are therefore strongly needed.

Here, we applied an in-house ddPCR-based assay for SARS-CoV-2 detection in rtPCR negative swab samples. Thanks to this RdRp-based assay, SARS-CoV-2 genome could be accurately identified and quantified down to only 2.9 (95% IC 2.0–11.5) copies per reaction corresponding to a viral load of 28 (95% IC 13–118) copies/mL, highlighting the great sensitivity of the method. Moreover, being the quantification per mL highly dependent on the quality of sampling and the extraction procedure, the presence in this assay of an internal reference gene (RNAseP) as a quality control, excludes any eventual PCR inhibition and confirms successful RNA extraction, thus dramatically reducing the risk of false negative results. RNAseP showed a quantification close to 10^5^ copies/mL in the majority of samples, with few peaks of 10^6^, suggesting a relatively stable expression of this gene in our population. Of note, when the SARS-CoV-2 amount was normalized according to the RNAseP quantification (per 100,000 copies of RNAseP) viral load results did not differ significantly respect to quantifications per mL in the majority of patients (73.6%, 14/19, S1 Table). A 1 log decrease was observed only for 5 samples, all characterized by an RNAseP expression at least 0.5 log higher respect to the mean value (S1 Table).

By using this reliable, reproducible and highly sensitive assay, we could improve the efficiency of COVID-19 diagnosis on nasopharyngeal swabs even at a very early stage of viral replication, or in anatomical districts where the virus is present at lower levels, such as the oropharyngeal or the nasopharyngeal samples [8, 9]. This bridges a significant diagnostic gap left by commercial rtPCR systems used to date as a bulwark of molecular diagnostics of SARS-CoV-2. This provides a significant clinical advantage in terms of prompt identification of subjects with SARS-CoV-2 infection, and for the consequent implementation of all appropriate containment and therapeutic measures. Indeed, all the 19 patients tested positive by ddPCR were characterized by generally low viral loads (never above the 2,000 copies/mL) that potentially justified the negative contextual rtPCR results. The SARS-CoV-2 detection by ddPCR in these patients was also confirmed by the presence of SARS-CoV-2 IgG at the later stage of illness (from 3 days to 90 days after, 14/14 patients with a serum sample available). On the other side, ddPCR did not detect SARS-CoV-2 genomes in 36 patients hospitalized during the pandemic and for whom (out one, probably hospitalized at a late-stage disease, when the viral clearance was already occurred) subsequent serological assays excluded a previously contact with SARS-CoV-2.

Taken together, our results support the superiority of our ddPCR-based assay to currently used rtPCR tests for the molecular detection of SARS-CoV-2.

The ability of ddPCR to generate accurate quantitative data has had a huge impact on the study of viral agents of infectious disease [8, 10–12, 14, 18]. Thanks to an extremely high sensitivity, this next generation PCR platform is providing a consistent help in all those clinical scenarios in which is of great importance to identify even the smallest amount of viral particles [10,12,14]. In the context of COVID-19 diagnosis, two recent studies highlight the best performances of ddPCR in detecting low viral load samples [19,20]. In particular, by targeting ORF1ab and N genes, these papers demonstrated that negative rtPCR samples could be identified as positive by the optimized ddPCR (4/96 samples in [19] and 25/27 samples in [20]). In the Suo et al paper, the SARS-CoV-2 positivity by ddPCR was confirmed by rtPCR in subsequent follow-up investigations for all the 25 patients [19]. Differently, our study showed the ddPCR advantages over currently available rtPCR approaches also in the setting of persistently and repeatedly negative rtPCR results. In particular, without this assay, no molecular laboratory confirmation of SARS-CoV-2 would have been available at hospitalization for 12 patients, all characterized by a severe COVID-19 manifestation and with evidence of bilateral interstitial pneumonia, and for two patients affected by pneumonia with pleuritis (n = 1), and lung cancer (n = 1), for whom the mere clinical evaluation excluded the COVID-19.

Our study has some limitations. a) The sample size was small (the study has been designed as a proof of concept, though); b) The correlation of the SARS-CoV-2 viral load with the timing of infection was not feasible; c) The infectivity of the virus was not assessed.

Moreover, the ddPCR turnaround time (longer than for qPCR), and the difficulty to convert this system in a high throughput individual assay makes it necessary to apply this system only in specific clinical scenarios. As this assay could potentially be used on different samples, the evaluation of its performances in different laboratories will be necessary for the inter-laboratory reproducibility, and thus the cross-validation of the methods.

Overall, ddPCR should be a complement to the current standard rtPCR-based diagnosis, in order to improve as much as possible the rapid identification of SARS-CoV-2 infection (making possible the diagnosis long before the viral load peak is reached and antibodies appear) and thus the definitive containment of the pandemics. Even more relevant, this test could be used for treatment monitoring, or for all supposed COVID-19 patients, who are negative for nasopharyngeal swab nucleic acid tests twice by rtPCR, but probably at risk of carrying SARS-CoV-2.

In conclusion, we have provided preliminary results to prove that ddPCR is a promising molecular diagnostic tool to detect low levels of SARS-CoV-2 RNA. It possesses all the critical characteristics that would allow its use to improve and accelerate COVID-19 diagnosis in clinical samples. As its use could be foreseen in routine clinical practice, additional data on a larger number of clinical samples, and possibly from multicenter studies, are required to further confirm its sensitivity, specificity, and reliability.

## Supporting information

S1 Fig **(A)** Amplification by digital droplet PCR of RdRp by serial diluition in the reference sample. **(B)** Amplification by digital droplet PCR of RdRp in negative controls. **(C)** Amplification by digital droplet PCR of RdRp in the sample 19. **(D)** Amplification by digital droplet PCR of RNAseP in the sample 19.
(PDF)

S1 Table Clinical characteristics of the 19 patients with a ddPCR positive nasopharyngeal swab sample at hospital admission. (DOCX)
