# Supplementary figures and images for: Detection and quantification of SARS-CoV-2 by droplet digital PCR in real-time PCR negative nasopharyngeal swabs from suspected COVID-19 patients
Source: PLoS One. 2020 Sep 8;15(9):e0236311. doi: 10.1371/journal.pone.0236311 (PMC7478621; doi:10.1371/journal.pone.0236311)

Supplementary Figure 1

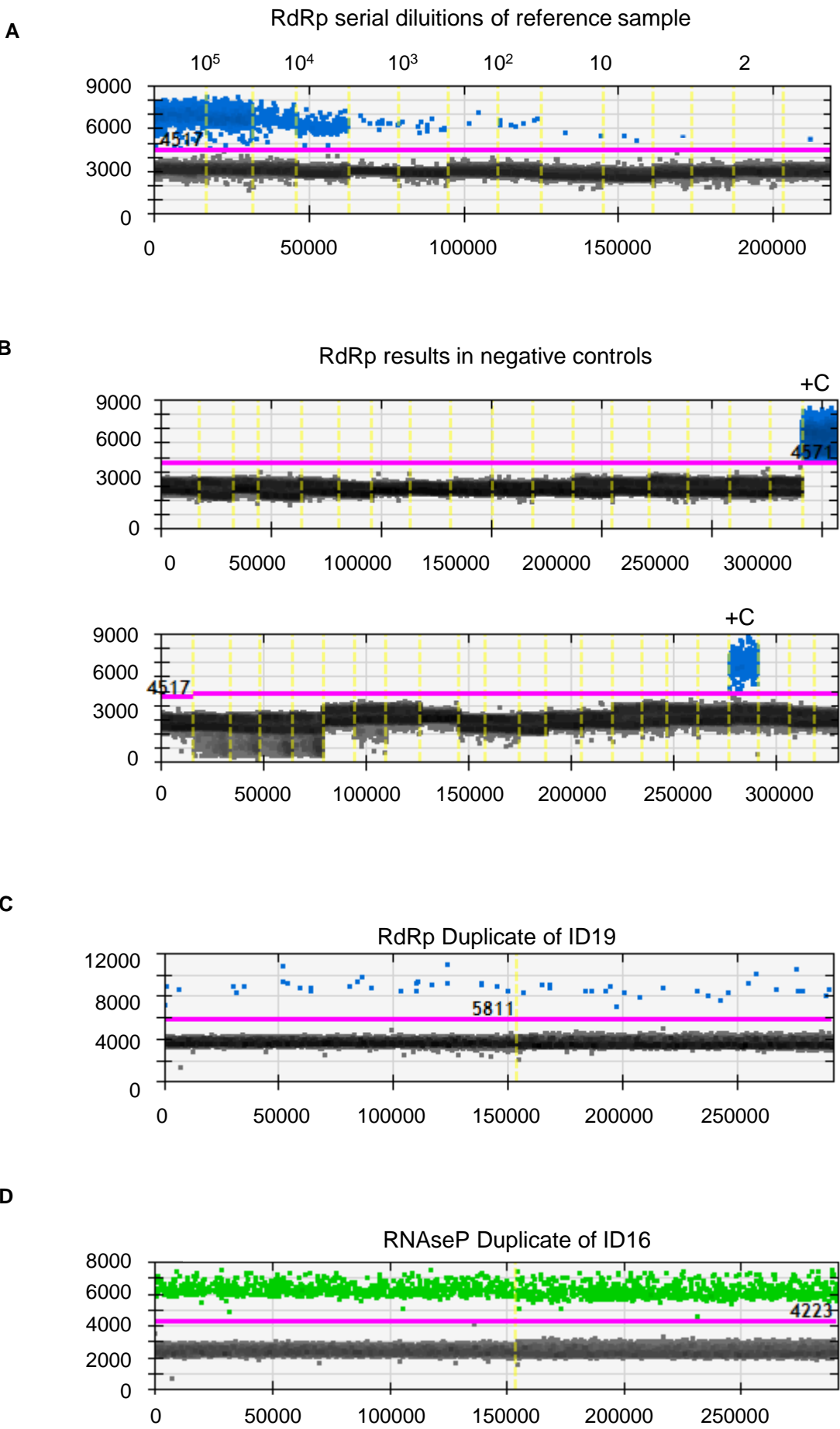

Supplement: S1 Fig — (A) Amplification by digital droplet PCR of RdRp by serial diluition in the reference sample. (B) Amplification by digital droplet PCR of RdRp in negative controls. (C) Amplification by digital droplet PCR of RdRp in the sample 19. (D) Amplification by digital droplet PCR of RNAseP in the sample 19. (PDF) [file pone.0236311.s001.pdf]
