# Supplementary material for: Detection and quantification of SARS-CoV-2 by droplet digital PCR in real-time PCR negative nasopharyngeal swabs from suspected COVID-19 patients
Source: PLoS One. 2020 Sep 8;15(9):e0236311. doi: 10.1371/journal.pone.0236311 (PMC7478621; doi:10.1371/journal.pone.0236311)
Supplement: S1 Table — (DOCX) [file pone.0236311.s002.docx]

**Supplementary Table 1: Clinical characteristics of the 19 patients with a ddPCR positive nasopharyngeal swab sample at hospital admission**

| **ID** | **Hospitalization date** | **Symptoms** | **Pulmonary involvement** | **COVID-19 typical presentation** | **Disease severity^a^** | **SARS-CoV-2 viral load at hospitalization** | | **IgG results^d^** |
| --- | --- | --- | --- | --- | --- | --- | --- | --- |
|  | **(month, year)** |  |  |  |  | **copies/mL^b^** | **copies/100,000 RNAseP^c^** |  |
| 1 | March, 2020 | Fever, Dyspnea | Bilateral interstitial pneumonia | yes | Severe | 30 | 58 | na |
| 2 | March, 2020 | Fever, Cough, | Lobar Pneumonia | yes | Moderate | 30 | 21 | + in a serum sample collected 3 days after the swab |
| 3 | March, 2020 | Fever, Cough, Dyspnea | Bilateral interstitial pneumonia | yes | Severe | 49 | 30 | + in a serum sample collected 80 days after the swab |
| 4 | March, 2020 | Fever, Dyspnea | Bilateral interstitial pneumonia | yes | Moderate | 56 | 44 | na |
| 5 | April, 2020 | Fever, Dyspnea | Bilateral interstitial pneumonia | yes | Severe | 60 | 50 | + in a serum sample collected 15 days after the swab |
| 6 | April, 2020 | Fever, Cough, Dyspnea | Bilateral interstitial pneumonia | yes | Severe | 84 | 47 | + in a serum sample collected 16 days after the swab |
| 7 | March, 2020 | Fever, Cough, Dyspnea | Bilateral interstitial pneumonia | yes | Severe | 111 | 8 | + in a serum sample collected 22 days after the swab |
| 8 | March, 2020 | none | none | no | Asymtomatic/Mild^e^ | 120 | 16 | na |
| 9 | April, 2020 | Fever, Cough, Dyspnea | Bilateral interstitial pneumonia | yes | Severe | 120 | 107 | + in a serum sample collected 27 days after the swab |
| 10 | March, 2020 | Fever, Cough, Dyspnea | Bilateral interstitial pneumonia | yes | Moderate | 128 | 25 | + in a serum sample collected 3 days after the swab |
| 11 | March, 2020 | Fever, Dyspnea | Pneumonia with pleuritis | no | Severe | 163 | 16 | + in a serum sample collected 24 days after the swab |
| 12 | May, 2020 | Dyspnea | Lung cancer | no | Severe | 207 | 40 | na |
| 13 | April, 2020 | Fever, Dyspnea | Bilateral interstitial pneumonia | yes | Severe | 280 | 110 | na |
| 14 | April, 2020 | Fever, Cough, Dyspnea | Bilateral interstitial pneumonia | yes | Severe | 300 | 459 | + in a serum sample collected 31 days after the swab |
| 15 | March, 2020 | Cough, Dyspnea | Bilateral interstitial pneumonia | yes | Severe | 390 | 558 | + in a serum sample collected 3 days after the swab |
| 16 | March, 2020 | Fever, Coght, Dyspnea | Bilateral interstitial pneumonia | yes | Moderate | 411 | 558 | + in a serum sample collected 10 days after the swab |
| 17 | March, 2020 | Fever, Cough, Dyspnea | Bilateral interstitial pneumonia | yes | Severe | 441 | 356 | + in a serum sample collected 3 days after the swab |
| 18 | March, 2020 | Fever, Dyspnea | Bilateral interstitial pneumonia | yes | Severe | 604 | 293 | + in a serum sample collected 17 days after the swab |
| 19 | March, 2020 | Cough, Dyspnea | Bilateral interstitial pneumonia | yes | Severe | 1800 | 3136 | + in a serum sample collected 3 days after the swab |

^a^The severity of the COVID-19 was classified into mild/asymptomatic, moderate, or severe, if showing i) mild clinical symptoms without sign of pneumonia on imaging, ii) fever and respiratory symptoms with radiological findings of pneumonia, iii) respiratory distress, with oxygen saturation ≤93% at rest, mechanical ventilation, or presence of multiorgan failure (septic shock) and/or admission to intensive care unit (ICU) hospitalization.

^b^Sensitivity threshold: 13 copies/mL.

^c^SARS-CoV-2 viral load was normalized according to RNAseP quantification.

^d^Chemiluminescent microparticle immunoassay IgG against SARS-CoV-2 (https://www.corelaboratory.abbott/us/en/offerings/segments/infectious-disease/sars-cov-2).The SARS-CoV-2 RNA positivity in samples with a serological assay not available (ID 1, 4, 8, 12, 13) was further confirmed by using a second assay adapted for ddPCR and targeting two different portions of RdRp (<https://www.who.int/docs/default-source/coronaviruse/real-time-rt-pcr-assays-for-the-detection-of-sars-cov-2-institut-pasteur-paris.pdf?sfvrsn=3662fcb6_2>). SARS-CoV-2 was detected at later time points by standard qualitative rtPCR for ID 1, 4, 8.

^e^COVID-19 contact; hospital admission for abdominal surgery.
